# Matrix Stiffness Regulates Mechanotransduction and Vascular Network Formation of hiPSC-Derived Endothelial Progenitors Encapsulated in 3D Hydrogels

**DOI:** 10.1101/2025.04.11.648340

**Published:** 2025-04-12

**Authors:** Jiwan Han, Kathleen N Halwachs, Toni West, Bryce V Larsen, Michael S Sacks, Adrianne Rosales, Janet Zoldan

## Abstract

The mechanical properties of the extracellular matrix (ECM), particularly stiffness, regulate endothelial progenitor responses during vascular development, yet their behavior in physiologically compliant matrices (<1 kPa) remains underexplored. Using norbornene-modified hyaluronic acid (NorHA) hydrogels with tunable stiffness (190-884 Pa), we investigated how hydrogel stiffness influences cell morphology, endothelial maturation, mechanotransduction, and microvascular network formation in human induced pluripotent stem cell-derived endothelial progenitors (hiPSC-EPs). Our findings reveal a stiffness-dependent tradeoff between mechanotransduction and vascular network formation. At intermediate stiffness (551 Pa), cells exhibited the greatest increase in endothelial marker CD31 expression and Yes-associated protein (YAP)/ transcriptional coactivator with PDZ-binding motif (TAZ) nuclear translocation, indicating enhanced mechanotransduction and endothelial maturation. However, this did not translate to superior plexus formation. Instead, the most compliant matrix (190 Pa) supported greater vascular connectivity, characterized by longer branches (~0.03/volume vs. 0.015 at 551 Pa) and enhanced actin remodeling. 3D cell contraction measurements revealed a 15.6-fold higher basal displacement in compliant hydrogels, suggesting that cell-generated forces and matrix deformability collectively drive vascular morphogenesis. Unlike prior studies focusing on pathological stiffness ranges (>10 kPa), our results emphasize that vascularization is not solely driven by the most mechanotransductive environment but rather by a balance of compliance, contractility, and cell-induced remodeling. These findings underscore the need to design hydrogels that provide sufficient mechanotransduction for endothelial maturation while maintaining compliance to support dynamic vascular morphogenesis. This work provides a mechanically tuned framework for optimizing microenvironments to balance endothelial differentiation and vascular network formation in tissue engineering and regenerative medicine.

## Full Text Availability

The license terms selected by the author(s) for this preprint version do not permit archiving in PMC. The full text is available from the preprint server.

